# A Review of Targeted Pulmonary Arterial Hypertension-Specific Pharmacotherapy

**DOI:** 10.3390/jcm5120114

**Published:** 2016-12-06

**Authors:** Ali Ataya, Jessica Cope, Hassan Alnuaimat

**Affiliations:** Pulmonary Hypertension Program, Division of Pulmonary, Critical Care, and Sleep Medicine, University of Florida, 1600 SW Archer Rd, M452, P.O. Box 100225, Gainesville, FL 32610, USA; aliataya@gmail.com (A.A.); copej@shands.ufl.edu (J.C.)

**Keywords:** pulmonary hypertension, pulmonary arterial hypertension, nitric oxide, soluble guanylate cyclase, endothelin, prostacyclin

## Abstract

Significant advances in the understanding of the pathophysiology of pulmonary arterial hypertension over the past two decades have led to the development of targeted therapies and improved patient outcomes. Currently, a broad armamentarium of pulmonary arterial hypertension-specific drugs exists to assist in the treatment of this complex disease state. In this manuscript, we provide a comprehensive review of the current Food and Drug Administration (FDA)-approved pulmonary arterial hypertension-specific therapies, and their supporting evidence for adults, targeting the nitric oxide, soluble guanylate cyclase, endothelin, and prostacyclin pathways.

## 1. Introduction

The primary treatment goals of managing patients with pulmonary arterial hypertension include alleviating pulmonary hypertension related symptoms, preventing disease progression, and improving patient quality of life and survival. The management of patients with pulmonary arterial hypertension includes a targeted pharmacotherapeutic approach with medications that impact three key physiologic pathways: nitric oxide, prostacyclin, and the endothelin-1 pathway. Combination oral therapy is often initiated upon diagnosis of pulmonary arterial hypertension, while the decision to initiate a prostacyclin is often dependent on the severity of the patient’s World Health Organization (WHO) functional class status, III or IV [1]. In this manuscript we will provide a review of the current US Food and Drug Administration (FDA)-approved adult pulmonary arterial hypertension-specific therapies and the evidence behind their use.

## 2. Nitric Oxide Pathway

### 2.1. Phosphodiesterase-5 Inhibitors

There are currently two Food and Drug Administration (FDA)-approved phosphodiesterase type 5 inhibitors for the treatment of pulmonary arterial hypertension: sildenafil and tadalafil. Phosphodiesterase type 5 inhibitors augment the pathogenesis of pulmonary arterial hypertension by inhibiting the enzyme phosphodiesterase type 5, which is responsible for breaking down cyclic guanosine monophosphate (cGMP). Preventing the breakdown of cGMP thus allows for pulmonary vasculature vasodilation [2].

#### 2.1.1. Sildenafil

Sildenafil is a phosphodiesterase type 5 inhibitor indicated for the treatment of pulmonary arterial hypertension in adults with WHO functional class II–III symptoms to improve exercise capacity and delay time to clinical worsening. The recommended adult dose of sildenafil is 20 mg orally three times daily [3]. Doses greater than 20 mg orally, three times daily, have not shown additional benefit [2]. Concomitant use of nitrates, in any form, is contraindicated due to the risk of hypotension [3,4].

When compared to placebo, sildenafil has been associated with improvement in the 6-min walk distance (6MWD) test, WHO functional class status, and mean pulmonary arterial pressure (Table 1) [2]. Two additional studies have observed sildenafil in combination with intravenous epoprostenol and oral bosentan. Sildenafil, when used in combination with epoprostenol, was associated with improvements in the 6MWD test and functional class status [5]. When used in combination with bosentan, there was no difference in the primary outcome, time to death, clinical worsening of pulmonary arterial hypertension, or hospitalization when compared to placebo. The 6MWD was improved at 16 weeks in the intervention group compared to placebo; however, there was no difference in improvement in functional class status between groups [6]. Common side effects among these clinical trials included headache, dyspepsia, nausea, and flushing [1,2,5,6].

#### 2.1.2. Tadalafil

Tadalafil, a phosphodiesterase type 5 inhibitor, gained FDA approval in 2003 and is indicated for patients with pulmonary arterial hypertension to improve exercise capacity. The recommended adult dose is 40 mg orally, once daily. Similar to sildenafil, concomitant use of nitrates, in any form, is contraindicated due to the risk of hypotension [7,8].

In clinical trials, tadalafil (40 mg daily) was associated with a significant improvement in the 6MWD test, and led to increased time to clinical worsening and improvement in cardiac index when compared to placebo [9]. Long-term safety and efficacy outcomes were established in a 52-week extension trial that showed 34% of patients receiving tadalafil 20 or 40 mg daily had improved functional class status at 68 weeks with minimal adverse effects [10]. Tadalafil, when used in combination with ambrisentan, has been associated with a decreased composite endpoint of death, hospitalization, disease progression, and/or unsatisfactory long-term clinical response [11]. A summary of the clinical trials on phosphodiesterase 5 inhibitors is summarized in Table 1.

### 2.2. Soluble Guanylate Cyclase Stimulator

#### Riociguat

Riociguat is a soluble guanylate cyclase stimulator indicated for the treatment of adults with chronic thromboembolic pulmonary hypertension after surgical treatment or inoperable disease to improve exercise capacity and WHO functional class, and in the treatment of pulmonary arterial hypertension to improve exercise capacity, improve WHO functional class and to delay time to clinical worsening. Riociguat has a unique mechanism of action in that it acts synergistically with endogenous nitric oxide and directly stimulates soluble guanylate cyclase independent of nitric oxide availability. When riociguat stimulates the nitric oxide pathway, generation of cyclic monophosphate results in vasodilation. That being said, the receipt of concomitant nitrates, nitric oxide donors, or phosphosdiesterase type 5 inhibitors is contraindicated due to the risk of profound hypotension. The recommended initial adult dose is 1 mg orally, three times daily, up-titrated in increments of 0.5 mg three times daily every two weeks as tolerated with a maximum recommended dose of 2.5 mg three times daily. Due to the potential for embryo-fetal toxicity, females may only receive riociguat via the Adempas Risk Evaluation and Mitigation Strategy (REMS) program [12].

The authors of CHEST-1 evaluated the use of riociguat in patients with chronic thromboembolic pulmonary hypertension (CTEPH) with inoperable disease or those experiencing recurrent pulmonary hypertension post pulmonary endarterectomy (Table 2). Patients were excluded if they had received an endothelin-receptor antagonist, prostacyclin analogue, phosphodiesterase type 5 inhibitor, or nitric oxide donor within the three months prior to enrollment. The majority of patients included were of WHO functional class II (31%) and III (64%) at baseline. Riociguat, in comparison to placebo, was associated with an improvement in the 6MWD test, a decrease in pulmonary vascular resistance and an improvement in WHO functional class in 33% of patients [13].

Aside from CTEPH, riociguat was also studied in patients with pulmonary arterial hypertension in the PATENT-1 trial, (Table 2). Patients could be included if they were receiving an endothelin-receptor antagonist or prostanoid (excluding parenteral therapy) at stable doses for at least 90 days. Patients with WHO functional class II (42%) and III (53%) were the most prevalent. Overall, patients receiving riociguat had an increased 6MWD test, decreased pulmonary vascular resistance, and improvement in WHO functional class. Common side effects noted among these trials included headache and dyspepsia [14].

## 3. Endothelin-1 Pathway

### 3.1. Endothelin Receptor Antagonists

Overexpression of endothelin-1, a vasoconstrictive peptide, is the result of the endothelial cell dysfunction observed in pulmonary arterial hypertension. Increased levels of endothelin-1 leads to a reduction in the synthesis of vasodilators including nitric oxide and prostacyclin, which leads to a further imbalance in the vasodilation and vasoconstriction homeostasis of the pulmonary artery resulting in endothelial cell hyperproliferation and fibrosis. Endothelin receptor antagonists block binding of endothelin-1 to the receptor, preventing overexpression of endothelin-1 in patients with pulmonary arterial hypertension [15]. There are currently three available FDA-approved endothelin-receptor antagonists, including bosentan, ambrisentan, and macitentan.

#### 3.1.1. Bosentan

Bosentan is a non-selective, endothelin receptor antagonist that received FDA approval in 2001. Bosentan was approved for the treatment of pulmonary arterial hypertension in patients of New York Heart Association (NYHA) classes II–IV to improve exercise ability and to decrease clinical worsening. It is recommended to initiate treatment at 62.5 mg orally twice daily in adult patients for four weeks and then increase the dose to 125 mg orally twice daily [16].

Clinical studies comparing bosentan to placebo have observed an improvement in cardiopulmonary hemodynamics, improvement in functional class, Borg Dyspnea Index, and time to clinical worsening, (Table 3) [17,18,19,20]. Adverse events included peripheral edema, headache, and elevated aminotransferases [19,20].

Bosentan carries a black box warning for hepatotoxicity due to the association of at least a three-fold increase in aminotransferases (AST and ALT) in 11% of patients. Bosentan is also likely to cause major birth defects if used by pregnant females based on available animal data. Due to the observed risks of hepatotoxicity and embryo-fetal toxicity, bosentan is available only through a restricted program called the Tracleer REMS Program. Under the Tracleer REMS program, prescribers, patients, and pharmacies must enroll in the program and patients must have monthly liver function tests drawn in addition to monthly pregnancy tests for females [16].

#### 3.1.2. Ambrisentan

Ambrisentan is a selective endothelin receptor A (ET_A_) antagonist that received FDA approval in 2007 for patients with pulmonary arterial hypertension NYHA functional class II–III. There are two endothelin receptor subtypes, ET_A_ and ET_B_, which mediate the effects of endothelin-1 on the pulmonary vasculature. The primary effects of ET_A_ are vasoconstriction and endothelial cell proliferation, while the actions of ET_B_ include vasodilation, antiproliferation, and endothelin-1 clearance. Ambrisentan has a 4000-fold increased selectivity for ET_A_ in comparison to ET_B_, however, the clinical significance of this is not known [21].

The adult ambrisentan dose is 5 or 10 mg once daily [21]. Ambrisentan also carries the risk of embryo-fetal toxicity. Female patients may only receive ambrisentan through the Letairis REMS program, with monitoring in the form of monthly pregnancy tests. Unlike bosentan, ambrisentan does not carry a black box warning for hepatotoxicity [21].

In clinical trials, ambrisentan led to an increased 6MWD, improvement in WHO functional class, time to clinical worsening and Borg Dyspnea Index, (Table 3) [22]. Patients receiving ambrisentan 10 mg orally daily were observed to have a greater improvement in the 6MWD test, suggesting a possible dose response with the 10 mg dose in comparison to the 2.5 and 5 mg dosing schemes. In combination with tadalafil, ambrisentan showed marked improvement in a primary composite endpoint of death, hospitalization for worsening pulmonary arterial hypertension, disease progression, and unsatisfactory long-term clinical response in comparison to ambrisentan and tadalafil monotherapy [11]. The AMBITION study supports initiating dual targeted therapy with ambrisentan and tadalafil as first-line management in patients with WHO functional class II–III pulmonary arterial hypertension [11].

#### 3.1.3. Macitentan

Like bosentan, macitentan is a nonselective, dual ET_A_ and ET_B_ receptor antagonist. Macitentan received FDA approval in 2013 for the treatment of pulmonary arterial hypertension, in patients with WHO FC I–III, to delay disease progression. The recommended adult dose of macitentan is 10 mg orally, daily. Similar to bosentan and ambrisentan, macitentan is also teratogenic and females are required to enroll in the Opsumit REMS Program [24].

The SERAPHIN study evaluated the clinical impact of macitentan in those patients with pulmonary arterial hypertension (PAH) WHO FC II–IV, (Table 3). It is noteworthy that approximately 60% of patients enrolled were already receiving a phosphodiesterase type 5 inhibitor at baseline. Initiation of macitentan resulted in a decrease in the time to worsening of pulmonary arterial hypertension. Adverse effects, in comparison to placebo, were significant for headache, nasal congestion and anemia [23].

## 4. Prostacyclin Pathway

The prostacyclin class of medications was first introduced with the advent of intravenous epoprostenol (Flolan^®^) in 1995. Additional compounds, including treprostinil and iloprost, subsequently gained FDA approval in various dosage forms including intravenous (Flolan^®^, Veletri^®^, and Remodulin^®^), subcutaneous (Remodulin^®^), oral (Orenitram^®^) and intermittent inhaled (Tyvaso^®^ and Ventavis^®^) [1]. The mechanism of action of prostacyclin analogues includes direct vasodilation of pulmonary and systemic arterial vascular beds, inhibition of platelet aggregation and anti-proliferative effects [25].

### 4.1. Parenteral Prostacyclin Therapies

Intravenous epoprostenol is approved for use in patients with pulmonary arterial hypertension WHO functional class III and IV. The recommended adult dose is 2 ng/kg/min up titrated by 1–2 ng/kg/min every 15 min or longer, based on dose-limiting side effects, to the maximally tolerated dose. Epoprostenol is administered via a continuous infusion due to its short half-life of three to five minutes. Two FDA-approved products are available including Flolan^®^ and Veletri^®^. Flolan^®^ is stable at room temperature for a maximum of 8 h, whereas Veletri^®^ is stable at room temperate for 24 to 48 h depending on the concentration. The pH of Flolan^®^ and Veletri^®^ are also slightly different, approximately 10.5 and 12, respectively. A new thermostable version of Flolan^®^ with a higher pH of 12 is currently being introduced to replace the non-thermostable product. Due to the pH, epoprostenol products must be delivered via a centrally inserted catheter. Common side effects include jaw pain, nausea, vomiting, diarrhea, headaches and complications related to drug-delivery system malfunction which have the potential to be life threatening [26,27].

In clinical trials, intravenous epoprostenol has been shown to reduce mortality, mean pulmonary arterial pressure, pulmonary vascular resistance, and increase exercise capacity and patient quality of life, (Table 4) [28,29]. Rare, but life-threatening, complications can occur secondary to drug-delivery system malfunction or catheter-related line infections [29]. Adequate training regarding aseptic technique, medication reconstitution, and available support must be established prior to discharging patients to the home setting on continuous prostacyclin therapy.

Treprostinil is a prostacyclin vasodilator indicated for the treatment of pulmonary arterial hypertension to improve exercise capacity in patients who are classified as WHO functional class II–IV. Remodulin^®^ first gained FDA approval in 2002 and can be administered both intravenously and subcutaneously as a continuous infusion. Treprostinil must be infused via central venous catheter if administered intravenously. Treprostinil has a biphasic, terminal half-life of approximately four hours. The recommended initial adult dose is 1.25 ng/kg/min to be up titrated by 1.25 ng/kg/min as tolerated. Side effects observed are similar to those seen with intravenous epoprostenol, including jaw pain, headaches, diarrhea, and nausea; however, unique to subcutaneous treprostinil is infusion site pain, which has led to discontinuation of therapy in some cases [30]. 

Treprostinil, when compared to placebo, has been associated with an increase in exercise capacity, improvement in indices of dyspnea, cardiopulmonary hemodynamics, WHO functional class status, and patient quality of life, (Table 4). Due to the longer half-life of treprostinil, in comparison to epoprostenol, there is a potentially decreased risk for rebound pulmonary hypertension due to drug-delivery system malfunction. Additionally, administering treprostinil subcutaneously avoids complications associated with indwelling central catheters including line occlusion and infections, which can be life threatening. However, subcutaneous treprostinil is associated with a high incidence of injection site pain and reactions that have led to discontinuation of therapy in approximately 10% of patients initiated on therapy in clinical trials [31,32].

### 4.2. Inhalational Prostacyclin Therapies

Two, FDA-approved, inhalational prostacyclin analogues are available for long-term use including iloprost (Ventavis^®^) and treprostinil (Tyvaso^®^). Both therapies are administered via intermittent inhalation via specific nebulizers unique to each medication. Iloprost gained FDA approval for use in patients with pulmonary arterial hypertension WHO functional class III–IV symptoms to improve exercise capacity, functional class, and decrease time to clinical deterioration. The recommended adult iloprost dose is 2.5 mcg nebulized six to nine times per day via the I-neb AAD System^®^ to be up-titrated to a maximum recommended dose of 5 mcg six to nine times daily as tolerated. Iloprost has a half-life of approximately 30 min. Side effects include flushing, cough, headache, and trismus [36]. Clinical trials indicate that long-term inhaled administration of iloprost improves exercise capacity, WHO functional class status, cardiopulmonary hemodynamics, and improves patient quality of life, (Table 4). In addition to the side effects mentioned above, a serious adverse effect, syncope, was noted in 5% of patients in a trial conducted by Olschewski and colleagues compared to 0% in those receiving placebo. The events occurred two to nine hours after the last inhalational dose of iloprost increasing the likelihood of syncope being related to the medication’s short half-life [33].

Inhaled treprostinil gained FDA approval in 2009 for use in patients with pulmonary arterial hypertension WHO functional class III symptoms to increase exercise capacity. The recommended adult dose is 3 breaths four times daily to be up titrated by 3 breaths as tolerated to a maximum recommended dose of 9 puffs four times day (54 mcg of treprostinil). Inhaled treprostinil must only be used with the Tyvaso Inhalational System, the Optineb-ir device. Common side effects include cough, headache, throat irritation, nausea, flushing and syncope. McLaughlin and colleagues studied the effects of inhaled treprostinil added on to oral pulmonary arterial hypertension therapies, including sildenafil and bosentan, (Table 4). The authors noted a significant improvement in 6MWD test in those receiving inhaled treprostinil in comparison to placebo as well as increased patient quality of life, however, no difference in the Borg Dyspnea Index or WHO functional class status was observed over the 12-week study period [34].

Inhalational prostacyclin therapies have potential advantages over parenteral prostacyclin in that they are less invasive and have less risk for complications such as line occlusion, infections, or drug-delivery system malfunction leading to rebound pulmonary hypertension. That being said, intravenous prostacyclins are still the only therapy to have shown a mortality benefit when compared to placebo [28,29,31,32,33,34].

Risk versus benefit must be weighed prior to initiation of therapy with all of the prostacyclins and individualized based on disease severity, patient competency, and side effect profile.

### 4.3. Oral Prostacyclin Therapy

Oral treprostinil (Orenitram^®^) was the first oral prostacyclin to gain FDA approval in 2013. Orenitram^®^ is indicated for the treatment of pulmonary arterial hypertension to improve exercise capacity in patients with WHO FC II–III symptoms. Oral treprostinil is an extended release tablet dosed 0.125 mg three times daily, taken approximately 8 h apart. It is recommended to increase the dose to the highest tolerated dose in increments of 0.125 mg orally three times daily. A maximum dose has not been defined and is dependent on patient tolerability. Common side effects include headache, diarrhea, nausea, flushing, and jaw pain. In clinical trials, oral treprostinil has been associated with an improved in 6MWD compared to placebo, however, all other clinical endpoints were not found to be significantly different between groups [35]. In two additional clinical trials, oral treprostinil was not shown to have additional benefit when added to other oral pulmonary arterial hypertension therapies including endothelin receptor antagonists and phosphodiesterase type 5 inhibitors over the course of 16 weeks, (Table 4) [37,38].

## 5. Selexipag

Selexipag is a novel oral prostacyclin receptor agonist that gained FDA approval in 2015 for the treatment of pulmonary arterial hypertension to delay disease progression and reduce the risk of hospitalization. Activation of the prostacyclin receptor results in an increased production of cyclic adenosine monophosphate, which allows for vascular smooth muscle relaxation. The recommended initial adult dose is 200 mcg orally, given twice daily to be increased in increments of 200 mcg twice daily at weekly intervals. The dose should be increased to the patient’s highest tolerated dose up to a maximum of 1600 mcg twice daily [39].

The GRIPHON study was a multicenter, double-blind randomized, parallel-group, placebo-controlled, event-driven phase three study that included patients aged 18–75 years who had idiopathic or heritable pulmonary arterial hypertension or pulmonary hypertension associated with human immunodeficiency virus (HIV), drug use or toxin exposure, connective tissue disease, or repaired congenital systemic-to-pulmonary shunts. Patients were randomized to receive selexipag 200 mcg twice daily or placebo. Dose titration occurred at weekly intervals until adverse effects where intolerable including headaches, jaw pain, nausea and vomiting [40].

A total of 1156 patients were enrolled from December 2009 through May 2013 and were followed for approximately 5.5 years. The majority of patients included were WHO functional class II (*n* = 529, 45.8%) and III (*n* = 607, 52.5%). Nine-hundred and twenty patients (79.6%) were receiving concurrent targeted PAH therapy at baseline including endothelin-receptor antagonist monotherapy (*n* = 170, 14.7%), phosphodiesterase type 5 inhibitor monotherapy (*n* = 374, 32.4%), and concomitant endothelin-receptor antagonists and phosphodiesterase type 5 inhibitors (*n* = 376, 32.5%). Overall, 397 (34.2%) patients had a primary end-point event which was defined as death or a complication of pulmonary arterial hypertension including disease progression or worsening of pulmonary arterial resulting in hospitalization, initiation of parenteral prostacyclin therapy or long-term oxygen therapy, or the need for lung transplantation or balloon atrial septostomy. Two hundred and forty-two patients (41.6%) in the placebo group and 155 (27%) patients in the selexipag group had a primary endpoint event (HR 0.6, 99% CI 0.46 to 0.78; *p* < 0.001). Disease progression and hospitalization accounted for 81.9% of the events. At week 26, there was no difference between groups regarding the proportion of patients whose functional class did not worsen, 74.9% and 77.8% in the placebo and selexipag groups, respectively. The most common adverse events associated with selexipag use were headache (*n* = 375, 65.2%), diarrhea (*n* = 244, 42.4%), nausea (*n* = 193, 33.6%), jaw pain (*n* = 148, 25.7%), and vomiting (*n* = 104, 18.1%) [40].

## 6. Conclusions

The last few decades have observed major advancements in the understanding of the pathophysiology of PAH that has led to the discovery and development of new therapies. Modulation of the different pathways involved in the pathogenesis of this devastating disease state including the nitric oxide, endothelin, and prostacyclin pathways have resulted in a significant improvement in the course of the disease. These therapies improve functional capacity and reduce disease progression; however, long-term prognosis remains poor for many of these patients. We hope that the recent advances in the understanding of molecular mechanisms will lead to the discovery of a new generation of drugs for pulmonary arterial hypertension therapy.

## Figures and Tables

**Table 1:** Phosphodiesterase Type 5 Inhibitors Clinical Studies.

Study	Population	Intervention	Primary Outcome	Secondary Outcomes	Adverse Effects
**Sildenafil (Revatio^®^)**					
Galie et al. [2]	PAH (idiopathic, CTD-associated, post repair of a congenital systemic-to-pulmonary shunt) WHO FC I–IV (*n* = 278), predominantly II (39%) and III (58%)	Sildenafil 20 mg (*n* = 69) vs. 40 mg (*n* = 68) vs. 80 mg (*n* = 71) vs. placebo (*n* = 70)	**6MWD at 12 weeks vs. placebo** 20 mg: 45 m (99% CI, 21–70; *p* < 0.001) 40 mg: 46 m (99% CI, 20–72; *p* < 0.001) 80 mg: 50 m (99% CI, 23–77; *p* < 0.001)	**Improvement in WHO FC vs. placebo** 20 mg: 21% (95% CI, 9–33; *p* = 0.003) 40 mg: 29% (95% CI, 16–42; *p* < 0.001) 80 mg: 35% (95% CI, 22–48; *p* < 0.001) **mPAP vs. placebo** 20 mg: −2.1 mmHg (−4.3 to 0; *p* = 0.04) 40 mg: −2.6 mmHg (−4.4 to −0.9; *p* = 0.01) 80 mg: −4.7 mmHg (−6.7 to −2.8; *p* < 0.001)	**20 mg:** headache (46%), dyspepsia (13%), flushing (10%) **40 mg:** headache (42%), dyspepsia (9%), flushing (9%) **80 mg:** headache (49%), dyspepsia (13%), flushing (15%)
Simonneau et al. [5]	PAH (idiopathic, heritable, associated with anorexigen use, CTD, or corrected congenital heart defect) WHO FC I–IV (*n* = 265), predominantly II (26%) and III (67%)	Sildenafil 20–80 mg oral three times daily + IV epoprostenol	**6MWD > 30 m** 1 year: 45% 2 years: 35% 3 years: 26%	**WHO FC, improved** 1 year: 29% 2 years: 23% 3 years: 18% **Survival** 1 year: 91% 2 years: 81% 3 years: 74%	Headache (64%), diarrhea (47%), dyspnea (45%), nausea (45%), fatigue (40%), dizziness (39%), upper respiratory tract infection (36%), and flushing (32%)
McLaughlin et al. [6]	PAH (idiopathic, heritable, associated with anorexigen use, CTD, or corrected congenital heart defect) WHO FC I–IV (*n* = 334), predominantly II (42%) and III (58%)	Sildenafil + bosentan vs. sildenafil + placebo	**Time to death, hospitalization, or clinical worsening** 42.8% vs. 51.4% (*p* = 0.25)	**6MWD at 16 weeks** +21.8 m, 95% CI 5.9–37.8; *p* = 0.01 **WHO FC, improved** 15.7% vs. 16%; *p* = 1	LFTs > 3 ULN 21.8% vs. 6.4%, bosentan vs. placebo
**Tadalafil (Adcirca^®^)**					
Galie et al. [9]	PAH (idiopathic, heritable, associated with anorexigen use, CTD, HIV, an atrial–septal defect, or corrected congenital heart defect) WHO FC I–IV (*n* = 405), predominantly II (32%) or III (65%)	Tadalafil 2.5–40 mg daily vs. placebo 53% of patients were receiving bosentan	**6MWD at 16 weeks** Tadalafil 40 mg: +33 m (95% CI 15–50; *p* = 0.0004)	**Clinical worsening** Tadalafil 40 mg vs. placebo: 5% vs. 16% (*p* = 0.038) **WHO FC, improved** No difference between groups **Cardiac index** Tadalafil 40 mg vs. placebo: 0.6 L/min/m^2^ (95% CI 0.1 to 1.6; *p* = 0.028)	**Tadalafil 40 mg** Headache (42%), dyspepsia (10%), flushing (13%), myalgias (14%)
Oudiz et al. [10]	PAH (idiopathic, heritable, associated with anorexigen use, CTD, HIV, an atrial-septal defect, or corrected congenital heart defect) WHO FC I–IV (*n* = 357), predominantly II (50%) or III (41%)	Long-term safety (52 weeks) and efficacy of tadalafil 20 mg (T20) and 40 mg (T40) daily	**WHO FC at 68 weeks, improved** T20: 34% T40: 34%	**Variables associated with time to clinical worsening** PAH duration (*p* = 0.04) Baseline 6MWD (*p* < 0.0001) Bosentan use (*p* = 0.04) Duration of bosentan use (*p* = 0.0002)	Headache (22%), diarrhea (13%), back pain (12%), and peripheral edema (12%)

6MWD test—6-min walk distance test; CTD—connective tissue disease; FC—functional class; HIV—human immunodeficiency virus; IV—intravenous; LFTs—liver function tests; mPAP—mean pulmonary arterial pressure; PAH—pulmonary arterial hypertension; ULN—upper limit of normal; WHO—World Health Organization.

**Table 2:** Riociguat (Adempas^®^) Clinical Studies.

Study	Population	Intervention	Primary Outcome	Secondary Outcomes	Adverse Effects
Ghofrani et al. [13]	Inoperable CTEPH or those with CTEPH and persistent PH after undergoing pulmonary endarterectomy (*n* = 261) WHO FC I–IV, predominantly II (31%) and III (64%)	Riociguat 2.5 mg TID vs. placebo	**6MWD at 16 weeks** 46 m, 95% CI 25 to 67; *p* < 0.001	**Riociguat vs. placebo** **PVR** −246 dyn^x^sec^x^cm^−5^ (95% CI, −303 to −190 *p* < 0.001) **WHO FC** 33% vs. 15% moved to a lower class (improved), 62% vs. 78% stayed the same, and 7% vs. 5% moved to a higher class (worsened)	Headache (25%), dizziness (23%), dyspepsia (18%), and nasopharyngitis (15%)
Ghofrani et al. [14]	PAH (idiopathic, familial, associated with CTD, congenital heart disease, portal hypertension with liver cirrhosis, or anorexigen use) WHO FC I–IV (*n* = 443), predominantly II (42%) and III (53%) 44% of patients were receiving an ERA, 6% were receiving a prostacyclin	Riociguat 2.5 mg TID vs. riociguat 1.5 mg TID vs. placebo	**6MWD at 12 weeks** Rio 2.5 mg vs. placebo: +36 m (95% CI, 20 to 52; *p* < 0.001)	**Riociguat 2.5 mg vs. placebo** **PVR** −226 dyn ^x^sec ^x^cm^−5^ (95% CI, −281 to −170; *p* < 0.001) **WHO FC** 21% vs. 14% moved to a lower class (improved), 76% vs. 71% stayed the same, and 4% vs. 14% moved to a higher class (worsened) **Time to clinical worsening** 1% vs. 6%; *p* = 0.005	Headache (27%), dyspepsia (19%), peripheral edema (17%), and hypotension (10%)

6MWD test—6-min walk distance test; CTD—connective tissue disease; CTEPH—chronic thromboembolic pulmonary hypertension; ERA—endothelin-receptor antagonist; FC—functional class; PAH—pulmonary arterial hypertension; PH— pulmonary hypertension; PVR—pulmonary vascular resistance; Rio—riociguat; TID—three times daily; WHO—World Health Organization.

**Table 3:** Endothelin Receptor Antagonists Clinical Studies.

Study	Population	Intervention	Primary Outcome	Secondary Outcomes	Adverse Effects
**Bosentan (Tracleer^®^)**					
Channick et al. [18]	IPAH and scleroderma-associated PAH WHO FC III or IV (*n* = 32)	Bosentan vs. placebo	**Bosentan 125 mg** 6MWD + 76 m (95% CI 12–139; *p* = 0.021)	**Bosentan 125 mg** CI + 1 L/min^−1^·m^−2^ (95% CI 0.6–1.4, *p* < 0.0001) PVR −415 dyn s cm^−5^ (95% CI −608 to −221; *p* = 0.0002)	Non-significant between groups Three withdrawals in the placebo group secondary to clinical worsening
Rubin et al. [19]	IPAH and CTD-associated PAH WHO FC III and IV (*n* = 213)	Bosentan vs. placebo	**Bosentan 125 and 250 mg** 6MWD + 44 m (95% CI 21–67; *p* < 0.001)	**Bosentan 125 and 250 mg** WHO FC improvement + 12% (95% CI −3 to 25) **Bosentan 125 mg** Borg Dyspnea Index −0.1 ± 0.2 Increased time to clinical worsening (*p* = 0.01) **Bosentan 250 mg** Borg Dyspnea Index −0.6 ± 0.2 Increased time to clinical worsening (*p* = 0.01)	Hepatic aminotransferase levels 8× ULN 125 mg Q12h (*n* = 2) 250 mg Q12h (*n* = 5)
Galie et al. [17]	Eisenmenger-associated PAH WHO FC III (*n* = 54)	Bosentan vs. placebo	**Bosentan 125 mg** SpO_2_ on room air + 1% (95% CI −0.7 to 2.8)	**Bosentan 125 mg** mPAP −5.5 (SE 2.25, *p* = 0.0363) 6MWD + 53.1 m (*p* = 0.008)	Peripheral edema (19%), headache (14%), palpitations (11%), one patient with hepatic aminotransferases > 5× ULN
Galie et al. [20]	PAH WHO FC II (*n* = 185)	Bosentan vs. placebo 15.7% of patients were receiving concurrent sildenafil	**Bosentan 125 mg** PVR −22.6% (95% CI −33.5 to −10; *p* < 0.0001)	**Bosentan 125 mg** 6MWD + 19.1 m (95% CI −3.6 to 41.8; *p* = 0.0758) Time to clinical worsening HR 0.227 (95% CI 0.65–0.798; *p* = 0.0114)	Abnormal LFTS 8% (bosentan) vs. 3% (placebo)
**Ambrisentan (Letairis^®^)**					
Galie et al. [22]	PAH (idiopathic, associated with CTD, HIV, or anorexigen use) WHO FC I–IV (*n* = 393), predominantly II (38%) and III (55%)	**ARIES-1** Ambrisentan 5 or 10 mg once daily vs. placebo **ARIES-2** Ambrisentan 2.5 or 5 mg PO once daily vs. placebo	**Mean placebo-corrected 6MWD at week 12** *ARIES-1* Ambrisentan 2.5 mg (32 m, 95% CI 2–63; *p* = 0.022) Ambrisentan 5 mg (59 m, 95% CI 30–89; *p* < 0.001) *ARIES-2* Ambrisentan 5 mg (31 m, 95% CI 3–59; *p* = 0.008) Ambrisentan 10 mg (51 m, 95% CI 27–76; *p* < 0.001)	**ARIES-1** (2.5 and 5 mg vs. placebo) Time to clinical worsening (*p* < 0.001) Improvement in WHO FC (*p* = 0.036) Borg dyspnea score (−0.6, 95% CI −1.2 to 0; *p* = 0.017) **ARIES-2** (5 and 10 mg vs. placebo) Time to clinical worsening (*p* = 0.307) Improvement in WHO FC (*p* = 0.117) Borg dyspnea score (−1.1, 95% CI −1.8 to −0.4; *p* = 0.019)	Peripheral edema (17.2%), headache (18.4%), and nasal congestion (5.7%) No patients receiving ambrisentan developed aminotransferase concentrations > 3 ULN compared to 3 patients (2.3%) in the placebo group
Galie et al. [11]	PAH (idiopathic, hereditary, CTD associated, drugs or toxins, HIV) WHO FC II–III (*n* = 500)	Ambrisentan + tadalafil (combination group), ambrisentan monotherapy, and tadalafil monotherapy	**Death, hospitalization, disease progression, or unsatisfactory long-term clinical response** Combination (*n* = 46, 18%) Ambrisentan (*n* = 43, 34%) Tadalafil (*n* = 34, 38%) HR 0.5 (95% CI 0.35–0.72; *p* < 0.001) for the combination group vs. monotherapy	**Combination vs. monotherapy** NT-proBNP −67.2 (*p* < 0.001) Satisfactory clinical response at week 24 (1.56, 95% CI 1.05–2.32; *p* = 0.03) 6MWD + 49.98 m (*p* < 0.001) Improvement in WHO FC 33%–37% across all groups	**Combination therapy** Peripheral edema (45%), headache (42%), and nasal congestion (21%)
**Macitentan (Opsumit^®^)**					
Pulido et al. [23]	PAH (idiopathic or heritable, CTD-associated, repaired congenital systemic-to-pulmonary shunts, HIV, drug or toxin) WHO FC II–IV, age > 12 years (*n* = 742)	Macitentan 10 mg daily, 3 mg daily, or placebo 60% of patients were already receiving a PDE5-inhibitor at baseline	**Worsening of pulmonary hypertension or death** 10 mg (31.4%) vs. placebo (46.4%), HR 0.55 (95% CI 0.32–0.76; *p* < 0.001) 3 mg (38%) vs. placebo (46.4%), HR 0.70 (95% CI 0.52–0.96; *p* = 0.01)	**6MWD at 6 months** 10 mg vs. placebo: +22 m (97.5% CI, 3.2–40.8; *p* = 0.008) 3 mg vs. placebo: +16.8 m (97.5% CI, −2.7–36.4; *p* = 0.01) **Improvement in WHO FC** 10 mg vs. placebo: 22% vs. 13% (*p* = 0.006) 3 mg vs. placebo: 20% vs. 13% (*p* = 0.04)	Incidence of peripheral edema (17.1%) and hepatotoxicity (3.5%) were similar across all three groups Headache (13.6%) and anemia (13.2%) were more prevalent in those patients receiving 10 mg daily

6MWD—six-min walk distance; CI—cardiac index; CTD—connective tissue diseases; FC—functional class; HR—heart rate; IPAH—idiopathic pulmonary arterial hypertension; m—meters; LFTs—liver function tests; NT-proBNP—N-terminal pro b-type natriuretic peptide; PAH—pulmonary arterial hypertension; PDE5—phosphodiesterase type 5-inhibitor; PVR—pulmonary vascular resistance; ULN—upper limit of normal; WHO—World Health Organization.

**Table 4:** Prostacyclin Clinical Trials.

Study	Population	Intervention	Primary Outcome	Secondary Outcomes	Adverse Effects
**Parenteral (i.e., intravenous, subcutaneous)**					
Barst et al. [28]	PAH WHO FC III (*n* = 60, 74%) and IV (*n* = 21, 26%)	IV epoprostenol plus conventional therapy vs. conventional therapy **Conventional therapy** Anticoagulants, oral vasodilators, diuretics, cardiac glycosides, supplemental oxygen	**6MWD at 12 weeks** +31 m vs. −29 m (*p* < 0.002)	**WHO FC, improved** 40% (*n* = 16) vs. 3% (*n* = 1); *p* < 0.02 **Change in mPAP** −8% vs. +3% (*p* < 0.002) **Change in PVR** −21% vs. +9% (*p* < 0.001) **Mortality at 12 weeks** Eight patients died in the conventional group (*p* = 0.003) **Improved QOL** with epoprostenol (*p* < 0.01)	Jaw pain, diarrhea, flushing, headaches, nausea, and vomiting Complications due to the delivery system (*n* = 26): occlusion, catheter-related sepsis and nonfatal thrombotic event
McLaughlin et al. [29]	PAH WHO FC III (*n* = 75; 46%) and IV (*n* = 87, 54%)	Registry of IV epoprostenol patients (*n* = 162)	**WHO FC at 17 ± 15 months, baseline FC III** 15.5% improved to FC I, 56.9% improved to FC II, 27.6% remained at FC III **WHO FC at 17 ± 15 months, baseline FC IV** 1.8% improved to FC I, 19.3% improved to FC II, 68.4% remained at FC III **Exercise time in seconds at 17 ± 15 months** 217 ± 192 to 432 ± 232 (*p* < 0.0001) **Survival, observed vs. expected** 1 year: 87.8% vs. 58.9% (*p* < 0.001) 2 years: 76.3% vs. 46.3% (*p* < 0.001) 3 years: 62.8% vs. 35.4% (*p* < 0.001)		Local infections from indwelling catheter (*n* = 119), 70 episodes of sepsis, 10 tunnel infections, and 72 instances where the catheter had to be replaced
Hiremath et al. [31]	PAH (sporadic, familial, HIV- or collagen vascular disease-associated) WHO FC III (*n* = 42, 95%) and IV (*n* = 2, 5%)	IV treprostinil (*n* = 30) vs. IV placebo (*n* = 14) Mean treprostinil dose at 12 weeks was 72 ng/kg/min	**6MWD at 12 weeks** +83 m (95% CI, 7–187; *p* = 0.008)	**Borg dyspnea score at 12 weeks** −2 (95% CI, 4–0, *p* = 0.023) **WHO FC at 12 weeks** Improvement of 1 class (95%, −1 to 0; *p* = 0.023)	Headache (50% vs. 14%), diarrhea (33% vs. 7%), pain in extremity (40% vs. 7%), and pain in jaw (27% vs. 0%)
Simonneau et al. [32]	PAH (idiopathic or associated with CTD or congenital systemic-to-pulmonary shunts) WHO FC II (*n* = 53, 11.3%), III (*n* = 382, 81.4%), IV (*n* = 34, 7.4%)	SC treprostinil (*n* = 233) vs. SC placebo (*n* = 236) Both groups received conventional therapy (oral vasodilators, anticoagulants, diuretics, and digitalis) Maximal allowable dose at 12 weeks was 22.5 ng/kg/min	**6MWD at 12 weeks** +12 m (95% CI, 4.4–27.6; *p* = 0.006)	**Borg dyspnea score** Treprostinil: 4.3 ± 0.2 (baseline) to 3.2 ± 0.2 (12 weeks) Placebo: 4.4 ± 0.2 (baseline) to 4.2 ± 0.2 (12 weeks) Difference between groups: *p* < 0.0001 **mPAP at 12 weeks** −2.3 ± 0.5 vs. 0.7 ± 0.6; *p* = 0.0003 **CI at 12 weeks** +0.12 ± 0.04 vs. −0.06 ± 0.04; *p* = 0.0001	Infusion site pain (85% vs. 27%, *p* < 0.0001), diarrhea (25% vs. 16%, *p* = 0.009), jaw pain (13% vs. 5%, *p* = 0.001) 18 (8%) patients discontinued treatment in the SC treprostinil group secondary to infusion site pain
**Nebulized**					
Olschewski et al. [33]	PAH (idiopathic, anorexigen and CTD-associated) and CTEPH WHO FC III (*n* = 119, 58.6%) and IV (*n* = 84, 41.4%)	Inhaled iloprost (*n* = 101) vs. placebo (*n* = 102)	Clinical response * at 12 weeks OR 3.97 (95% CI, 1.47–10.75; *p* = 0.007)	**6MWD at 12 weeks** +34.6 m (*p* = 0.004) **WHO FC, improved by one class** 23.8% vs. 12.7% (*p* = 0.03) **mPAP at 12 weeks** −4.6 ± 9.3 vs. −0.2 ± 6.9 (*p* < 0.001) **PVR at 12 weeks** −239 ± 279 vs. +96 ± 322 (*p* < 0.001)	Syncope (5% vs. 0%, *p* = 0.03), cough (38.6% vs. 25.5%, *p* = 0.05), flushing (26.7% vs. 8.8%, *p* = 0.001), and jaw pain (11.9% vs. 2.9%, *p* = 0.02)
McLaughlin et al. [34]	PAH (idiopathic, familial, or collagen vascular disease-, HIV-, and anorexigen use-associated) WHO FC III (*n* = 230, 97.9%) and IV (*n* = 5, 2.1%) Patients could be receiving bosentan (*n* = 165, 70.2%) or sildenafil (*n* = 70, 29.8%) at a stable dose for at least three months prior to enrollment	Inhaled treprostinil vs. inhaled placebo Maximum dose: 9 puffs four times daily	**6MWD at 12 weeks** 20 m (95% CI, 8–32.8; *p* = 0.0004)	**Borg dyspnea score, WHO FC, PAH signs and symptoms** No difference between groups **QOL at week 12** Significantly increased with treprostinil (*p* = 0.027)	Cough (54% vs. 29%, *p* < 0.05), headache (41% vs. 23%, *p* < 0.05), and flushing (15% vs. <1%, *p* < 0.05)
**Oral**					
Jing et al. [35]	PAH (idiopathic, hereditable, anorexigen, collagen vascular disease-, HIV-, and congenital systemic-to-pulmonary shunt-associated) WHO FC II (*n* = 125, 35.8%) and III (*n* = 212, 60.7%)	Oral treprostinil (*n* = 233) vs. placebo (*n* = 116)	**6MWD at week 12** 23 m (95% CI, 4–41; *p* = 0.0307)	**Borg dyspnea score, WHO FC, or symptoms of PAH at week 12** No significant difference between groups	Headache (69% vs. 31%, *p* < 0.05), nausea (39% vs. 22%, *p* < 0.05), diarrhea (37% vs. 18%, *p* < 0.05), jaw pain (25% vs. 7%, *p* < 0.05), and flushing (21% vs. 8%, *p* < 0.05)

*Clinical response was defined as (a) improvement in exercise ability (6-min walk test) by at least 10% versus baseline evaluated 30 min after dosing, (b) improvement by at least one New York Heart Association (NYHA) class versus baseline, and (c) no death or deterioration of pulmonary hypertension. 6MWD—six-min walk distance; CI—cardiac index; CTD—connective tissue diseases; CTEPH—chronic thromboembolic pulmonary hypertension; FC—functional class; m—meters; IV- intravenous; mPAP—mean pulmonary arterial pressure; OR—odds ratio; PAH—pulmonary arterial hypertension; PVR—pulmonary vascular resistance; QOL—quality of life; SC—subcutaneous; WHO—World Health Organization.
